# Renoprotective effect of the antioxidant curcumin: Recent findings^☆^

**DOI:** 10.1016/j.redox.2013.09.003

**Published:** 2013-09-17

**Authors:** Joyce Trujillo, Yolanda Irasema Chirino, Eduardo Molina-Jijón, Ana Cristina Andérica-Romero, Edilia Tapia, José Pedraza-Chaverrí

**Affiliations:** a Department of Biology, Facultad de Química, UNAM, Ciudad Universitaria, 04510 México, DF, Mexico; b Unidad de Biomedicina, Facultad de Estudios Superiores Iztacala, UNAM, 54059 Estado de México, Mexico; c Department of Physiology, Biophysics and Neuroscience, Center for Research and Advanced Studies, National Polytechnic Institute (Cinvestav-IPN), 07360 México, DF, Mexico; d Laboratory of Renal Pathophysiology, Department of Nephrology, Instituto Nacional de Cardiología “Ignacio Chávez”, 14080 México, DF, Mexico

**Keywords:** Oxidative stress, Bifunctional antioxidant, Nrf2, Mitochondrial dysfunction, Renal hemodynamics, Nephrotoxicity

## Abstract

For years, there have been studies based on the use of natural compounds plant-derived as potential therapeutic agents for various diseases in humans. Curcumin is a phenolic compound extracted from *Curcuma longa* rhizome commonly used in Asia as a spice, pigment and additive. In traditional medicine of India and China, curcumin is considered as a therapeutic agent used in several foods. Numerous studies have shown that curcumin has broad biological functions particularly antioxidant and antiinflammatory. In fact, it has been established that curcumin is a bifunctional antioxidant; it exerts antioxidant activity in a direct and an indirect way by scavenging reactive oxygen species and inducing an antioxidant response, respectively. The renoprotective effect of curcumin has been evaluated in several experimental models including diabetic nephropathy, chronic renal failure, ischemia and reperfusion and nephrotoxicity induced by compounds such as gentamicin, adriamycin, chloroquine, iron nitrilotriacetate, sodium fluoride, hexavalent chromium and cisplatin. It has been shown recently in a model of chronic renal failure that curcumin exerts a therapeutic effect; in fact it reverts not only systemic alterations but also glomerular hemodynamic changes. Another recent finding shows that the renoprotective effect of curcumin is associated to preservation of function and redox balance of mitochondria. Taking together, these studies attribute the protective effect of curcumin in the kidney to the induction of the master regulator of antioxidant response nuclear factor erythroid-derived 2 (Nrf2), inhibition of mitochondrial dysfunction, attenuation of inflammatory response, preservation of antioxidant enzymes and prevention of oxidative stress. The information presented in this paper identifies curcumin as a promising renoprotective molecule against renal injury.

## Highlights

• Curcumin prevents mitochondrial dysfunction in nephrotoxicity.
• Curcumin prevents renal hemodynamic alterations in chronic renal failure.
• Curcumin is a therapeutic agent in chronic renal failure.
• Curcumin induces renal Nrf2 translocation.
• Curcumin is an antiinflammatory agent in renal injury.

## Introduction

### History and cultivation of *Curcuma longa*

*Curcuma longa* (turmeric or curcuma) is a rhizomatus monocotyledonous perennial herbaceous plant member of the ginger family (Zingiberaceae), endemic and prevalent in tropical and subtropical regions including India, China and South East Asia. India is the most important producer, consumer and exporter of turmeric. Its Latin name Curcuma, is derived from the Arabic word, Kourkoum, the original name for saffron [16]. *C. longa* and its growth requires a hot, humid climate with temperatures between 20 and 30 °C and great amounts of water [29]. Turmeric has long been known as a spice, remedy and dye, and since 1280, Marco Polo mentioned turmeric in his travel around China and India. In the 13th century, Arabian merchants brought turmeric to the European market from India. During the British settlement of India in the 15th century, turmeric was combined with several other spices to form curry powder.

### Curcuminoids and curcumin

**Fig. 1:**
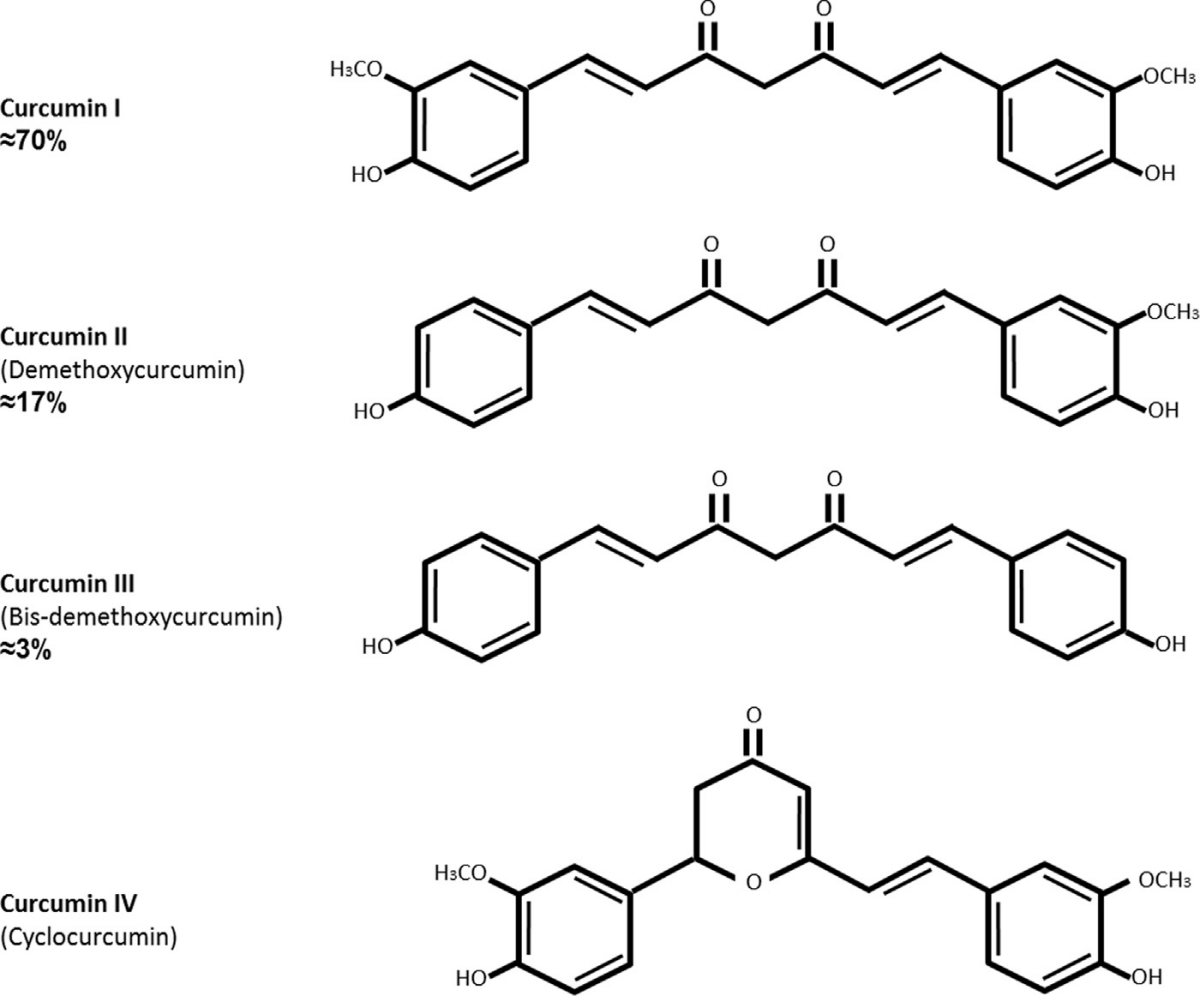
Chemical structures and abundance of curcuminoids in turmeric that have terapeutic effects.

Curcuma contains 60–70% carbohydrate, 8.6% protein, 5–10% fat, 2–7% fiber, 3–5% curcuminoids (50–70% curcumin) and up to 5% essential oils and resins. The curcuminoid content in turmeric may vary between 2 and 9%, depending on geographical conditions [29]. The composition of curcuminoids is approximately 70% curcumin (curcumin I), 17% demethoxycurcumin (curcumin II), 3% *bis*-demethoxycurcumin (curcumin III) and the rest (10%) is called cyclocurcumin (curcumin IV) [5,8,36] (Fig. 1). However, the last compound has been associated with poor or non-biological activity [61]. The most active component of turmeric is curcumin [67]; Vogel first isolated it in 1815 [29]. Curcumin is an orange–yellow crystalline powder practically insoluble in water. The structure of curcumin (Fig. 1) was first described in 1910 by Lampe and Milobedeska and proved to be diferuloylmethane [29]. Studies indicate that functional groups associated to curcumin chemical structure including *bis*-α,β−unsaturated β-diketone, two methoxy groups, two phenolic hydroxy groups and two double-conjugated bonds might play an essential role in antiproliferative and anti-inflammatory activities assigned to curcumin [3]. Curcumin has keto-enol tautomers, of which keto form predominates in acid and neutral solutions and enol form in alkaline solutions.

### Curcumin as a food additive

Turmeric is an ingredient of spice blends, mainly curry powder, which generally consists of turmeric, clove, paprika, ginger, cardamom, coriander, cumin, mace, pepper and cinnamon. Turmeric is commonly used as natural pigment (yellow 3) in the cosmetic and textile production but it is widely used in food industry. Indeed, turmeric is classified as an additive in E100 category and is used as food color additive in mustard, pastries, daily products and canned fish [40]. Furthermore, according to the Joint FAO/WHO Expert Committee on Food Additives (JECFA) the admissible daily intake (ADI) is 0–3 mg/kg body weight and it was established at the 61st JECFA in 2003 [29].

### Curcumin: Traditional uses in folk medicine and biological properties

Curcuminoids have been consumed as therapeutic infusions over the centuries worldwide. In Ayurvedic medicine, curcumin is a well-documented treatment for various respiratory conditions such as, asthma, bronchial hyperactivity and allergy, as well as for liver disorders, anorexia, rheumatism, diabetic wounds, runny nose, cough and sinusitis [10]. In traditional Chinese medicine curcumin has been used to treat diseases associated with abdominal pain [35]. In ancient Hindu medicine, it was used to treat sprains and swelling [10]. In Oriental cultures, it has traditionally been used as good therapeutic alternative, particularly as an anti-inflammatory, antioxidant, anticarcinogenic and antimicrobial reagent. In fact, it has scientifically proven that curcumin is indeed antioxidant [11,31,26,17], anti-inflammatory [2,82,86], and antibacterial [52]. Moreover, it has also been used because of its hepatoprotective [56,32,86], thrombosuppressive, neuroprotective [85,18,63], cardioprotective [37,23,73], antineoplasic [4,17,68], antiproliferative [12], hypoglycemic and antiarthritic effect [35]. Curcumin has also been used for the treatment of intestinal parasites and as a remedy for poisoning, snakebites and various other complaints [49]. In this review we are focused on the renoprotective effects of curcumin and the mechanisms involved in this effect.

### Antioxidant properties of curcumin

Oxidative stress plays a major role in the pathogenesis of various diseases including myocardial ischemia, cerebral brain ischemia-reperfusion injury, hemorrhage and shock, neuronal cell injury, hypoxia and cancer. Curcuminoids exhibit a differential antioxidant activity in several *in vitro* and *in vivo* models, for example, preventing lipid peroxidation in a variety of cells such as erythrocytes, rat brain homogenates, rat liver microsomes, liposomes and macrophages, where peroxidation is induced by Fenton's reagent, as well as for metals, hydrogen peroxide (H_2_O_2_) and 2,2′-azo-*bis*(2-amidino-propane) hydrochloride (AAPH) [62].

Furthermore, it has been reported that curcumin is a bifunctional antioxidant [26] because of its ability to react directly with reactive species and to induce an up-regulation of various cytoprotective and antioxidant proteins. Curcumin is able to scavenge superoxide anion (O_2_^¯∙^) [6,76], hydroxyl radicals (^∙^OH) [13], H_2_O_2_, [6,13], singlet oxygen [25], nitric oxide [77,78], peroxynitrite [44] and peroxyl radicals (ROO^∙^) [13]. Together, these mechanisms might explain, at least in part, some of the cytoprotective effects of this compound. Features as the presence of phenolic groups in the structure of curcumin (Fig. 1) explains its ability to react with reactive oxygen species (ROS) and reactive nitrogen species (RNS) and might probably be one of the mechanisms through which curcumin treatment protects the epithelial cells of renal tubules (LLC-PK1) from oxidative damage induced by H_2_O_2_ [22].

The indirect antioxidant capacity of curcumin is defined by its ability to induce the expression of cytoprotective proteins such as superoxide dismutase (SOD), catalase (CAT) [59], glutathione reductase (GR), glutathione peroxidase (GPx) [87], heme oxygenase 1 (HO-1) [42,63], glutathione-*S*-transferase (GST), NAD(P)H: quinone oxidoreductase 1 (NQO1) [88] and γ-glutamylcysteine ligase (γGCL) [65]. Furthermore, it has been reported that curcumin can increase the synthesis and concentration of reduced glutathione (GSH) in astrocytes and neurons by induction of γGCL [47]. The cytoprotective proteins induced by curcumin are regulated by the nuclear factor erythroid-derived 2 (Nrf2, [24,64]), which in turns is also activated by curcumin [17,28]. On the other hand, it is well known that encoding genes for cytoprotective proteins are induced coordinately by a common molecular mechanism in which the inductors highly modify reactive thiol groups of cysteine in the Kelch-Like ECH-Associated Protein 1 (Keap1) [27]. Keap1 protein is a zinc metalloprotein cysteine-rich bound to Nrf2 and normally associated with the protein complex cullin 3 (CuI3), which promotes the ubiquitination and subsequent proteosomal degradation, preventing Nrf2 translocation into the nucleus. Also, it was established that the gene expression of cytoprotective proteins is regulated by three cellular components: (i) Antioxidant response element (ARE) sequence, a specific sequence present in regulatory regions of the genes of cytoprotective proteins, (ii) Nrf2, a transcription factor consisting of a basic leucine zipper that regulates basal and inducible expression of cytoprotective genes, and (iii) Keap1, the chemical sensor for inductors. In general, the cysteine residues interaction of protein-Keap1 with some compounds induces conformational changes that abrogate the ability of Keap1 to repress Nrf2; this transcription factor migrates to the nucleus where it is combined with small Maf transcription factors. This complex binds to ARE facilitating the transcription of cytoprotective gene. By this reason, Nrf2 is considered a master regulator of the antioxidant response against oxidative stress.

### Renal diseases epidemiology

The homeostasis of body extracellular electrolyte composition and fluid volume is essential for all animals and humans to survive. The kidney plays a fundamental role in maintaining precise body and/or extracellular electrolyte, fluid balance and blood pressure homeostasis primarily through the actions of its proximal and distal tubular segments of nephrons [38]. Under renal insufficiency conditions, deregulation of extracellular electrolytes or overall fluids volume may lead to disturbance of the circulation, including cardiac output and blood pressure [39]. Prevalence of chronic kidney disease is estimated to be 8–16% worldwide [43] and it is expected that the number of patients with chronic kidney disease increase at a fastest rate in the poorest parts of the world. According to the 2010 Global Burden of Disease Study, chronic kidney disease was ranked 27th in the list of causes of total number of global deaths in 1990, but rose to 18th in 2010 [48]. Patients with chronic kidney disease are at an increase risk of acute kidney injury (AKI). Acute kidney injury might occur with the use of several drugs, such as non-esteroidal anti-inflammatory drugs, antibiotics, antineoplastic drugs, and angiotensin-converting-enzyme inhibitors. A meta-analysis of 13 cohort studies confirmed that AKI is an important risk factor for chronic and end-stage renal disease [21]. Severe, long, and repeated episodes of acute kidney injury increase the risk of progression of chronic kidney disease. Acute and chronic renal failures are global public health issues with different features to take into account in different parts of the world, renal complications, which involve most organ systems, can be treated and prevented, by using different therapeutic strategies.

## Renoprotective effect of curcumin

### Renal injury induced by diabetes

Diabetic nephropathy (DN) is one of the main causes of end-stage renal disease. DN is characterized by the presence of hyperfiltration, glomerular hypertrophy, tubular albuminuria, mesangial matrix expansion, and increased expression of extracellular matrix proteins that involves several profibrotic factors such as transforming growth factor β (TGF-β) and connective tissue growth factor (CTGF). The effect of curcumin on diabetic nephropathy has been studied [75]. Sharma et al. [69] have been found that curcumin administration (15 and 30 mg/kg/day for two weeks) protects against streptozotocin-induced diabetic nephropathy and oxidative stress. Furthermore, Soetikno et al. [72] evaluated in a diabetic nephropathy model the effect of oral curcumin administration (100 mg/kg/day for 8 weeks) showing that curcumin prevents progression of renal disease.

Curcumin treatment attenuates proteinuria and improves creatinine clearance after 3 weeks of streptozotocin injection. In addition, it decreased oxidative stress by reducing levels of subunits of nicotinamide adenine dinucleotide phosphate (NADPH) oxidase Nox4 and p67phox, which catalyzes the synthesis of O_2_^−∙^. Moreover, it also increased the activity of the antioxidant enzyme GPx.

The renoprotective effect of curcumin was related to the downregulation of the profibrotic cytokines vascular endothelial growth factor (VEGF), TGF-β, CTGF and osteopontin as well as in extracellular matrix proteins fibronectin and collagen IV. Moreover, it was observed a reduction in the development of structural damage evidenced by lower glomerulosclerosis index (GS), tubulointerstitial (IT) fibrosis and arteriolopathy. These effects might be in part mediated by inhibition of protein kinase C-β (PKC-β), kinase responsible for the phosphorylation of a wide diversity of proteins [72]. Cellular events such as inhibition of nuclear factor kappa-light-chain-enhancer of activated B cells (NF-κB) [20,71] and decrease of macrophage infiltration [71], histone acetyltransferase p300 protein and oxidative stress [20], also have been involved in the mechanisms through curcumin protects against diabetic nephropathy. Furthermore, the protective effect of curcumin in diabetic nephropathy has also been associated to the prevention of kidney triglycerides buildup [74]. Interestingly, curcumin also mitigates cardiac [73] and cerebral [46] complications in streptozotocin-induced diabetes.

Curcumin derivatives have also been proved to be effective in ameliorating diabetic nephropathy. Pan et al. [58,57] studied the effect of curcumin B06 and C66 analogues in diabetic rats (0.2, 1 and 5 mg/kg/day for 6 weeks) [57,58]. B06 treatment reduced the inflammatory kidney response by the attenuation of (a) renal macrophage infiltration, (b) expression of the profibrotic cytokine TGF-β, inducible nitric oxide synthase (iNOS), and cyclooxygenase-2 (COX-2) and (c) the proinflammatory cytokines such as tumor necrosis factor-alpha (TNF-α) and monocyte chemoattractant protein-1 (MCP-1). The anti-inflammatory effect of B06 was associated with the inhibition of c-Jun N-terminal kinase (JNK)/NF-κB activation [58,57]. Similarly, the oral administration (80 mg/kg/day for 45 days) of tetrahydrocurcumin (THU), another curcumin derivative, attenuated the renal and hepatic dysfunction found in rats with diabetes induced by streptozotocin and nicotinamide [53].

### Renal injury induced by 5/6 nephrectomy

The study of chronic progressive renal injury in rats as the 5/6 nephrectomy (5/6NX), which involves the removal of 5/6 of the renal mass, is useful in the evaluation of strategies to reduce renal injury. This model is characterized by proteinuria, hypertension, proliferation of smooth muscle cells of the glomerular arterioles (arteriolopathy), IT inflammation and hemodynamic alterations in individual nephrons [79].

**Fig. 2:**
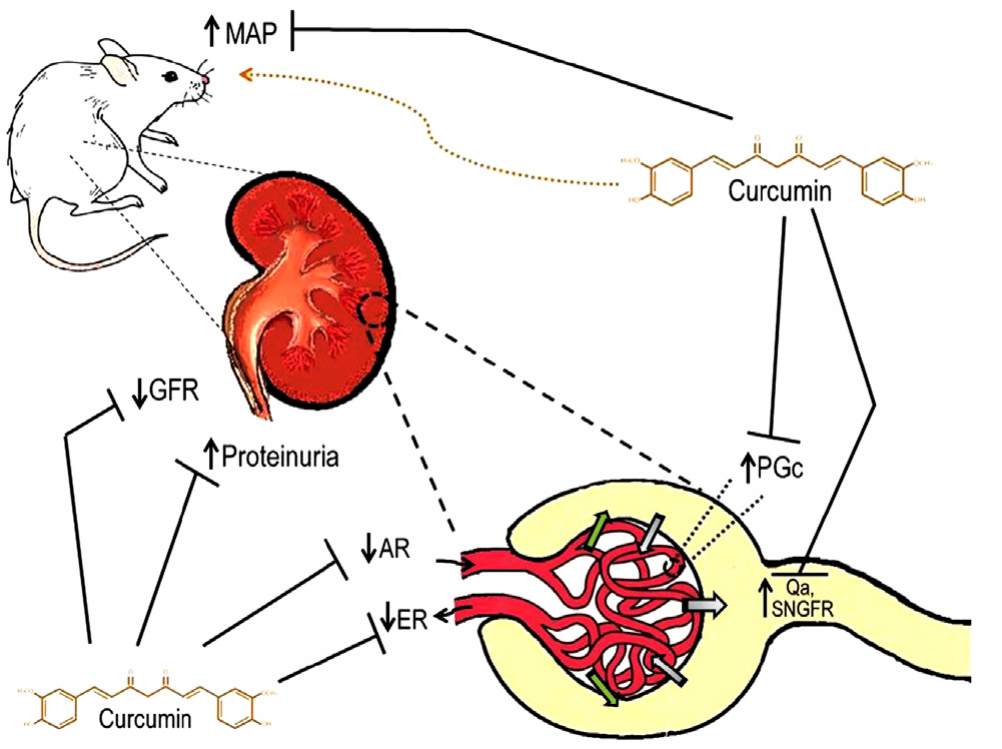
Curcumin prevents renal hemodynamic alterations. Curcumin ameliorates 5/6NX-induced alterations in mean arterial pressure (MAP), proteinuria and glomerular filtration rate (GFR) and in the following parameters of renal hemodynamics: single-nephron glomerular filtration rate (SNGFR), single-nephron plasma flow (Qa), glomerular capillary pressure (PGc), afferent resistance (AR) and efferent resistance (ER).

In 5/6NX renal injury model, Ghosh et al. [33] found that curcumin (75 mg/kg/day for 8 weeks) exerted a renoprotective effect associated with the attenuation of inflammation, macrophage infiltration and the high levels of TNF-α in plasma and kidney and also of renal NF-κB activation [33]. Further studies in this model by Tapia et al. [79] evaluated the renoprotective effect of curcumin (60 mg/kg for 37 days). Nephrectomized animals developed hypertension, proteinuria, increase in serum creatinine and blood urea nitrogen (BUN) and glomerular hemodynamic alterations including hyperfiltration (high single nephron glomerular filtration rate and single nephron glomerular plasma flow), glomerular hypertension (high glomerular capillary pressure) and decrease in the afferent and efferent resistances as well as renal injury characterized by GS and IT fibrosis and inflammation. Interestingly, after curcumin administration, all the above systemic and glomerular alterations were attenuated. This was the first work showing that curcumin is able to prevent glomerular hemodynamic alterations secondary to 5/6NX (Fig. 2). The protective effect of curcumin in this experimental model was associated to an increased activity of the antioxidant enzymes CAT, GPx, GR, GST and SOD and a decrease in oxidative stress. Additionally, Soetikno et al. [70] found that curcumin administration (75 mg/kg/day for 8 weeks) in 5/6NX rats could reduce proteinuria, systolic blood pressure, GS, IT damage and inflammatory markers as TGF-β, TNF-α, NF-κB and COX-2. They also found that curcumin attenuated malondialdehyde (MDA) levels (a lipid peroxidation marker) which was associated with lower expression of p67phox and p22phox, essential subunits of NADPH oxidase [70]. Both studies conducted by Tapia et al. [79,80] established that the protective mechanism achieved by curcumin in the kidney was mainly due to the nuclear translocation of Nrf2. Later, Tapia et al. [80] showed that curcumin postreatment (120 mg/kg for 30 days), given 30 days after the 5/6NX induction, was able to revert renal damage and oxidative stress. These experiments show that curcumin is a therapeutic agent in experimental chronic renal failure. It is important to highlight that tissue injury throwback effect of curcumin turns this molecule into a promising therapeutic agent. In this regard, it is also remarkable that curcumin can protect not only against renal injury but also against adverse effects derived from 5/6NX. For example, Correa et al. [23] showed that curcumin (120 mg/kg/day for 67 days) protects against cardiovascular disorders and cardiac tissue remodeling associated to the development of chronic renal failure in rats with 5/6NX. The cardioprotective effect of curcumin in 5/6NX model was associated with reduction in ROS production and oxidative stress markers, an increased antioxidant response and preservation of mitochondria function. Finally, Ghosh et al. [34] reported in this 5/6NX model that curcumin (75 mg/kg/day for 8 weeks) blocks overexpression of inflammatory mediators such as TNF-α and interleukin 1β (IL-1β) through activation of phospholipase 2 (PLP2) and COX-2, both key regulators of inflammation and oxidative stress inductors.

### Renal injury induced by ischemia and reperfusion (I/R) or by glomerulonephritis

The effect of curcumin on acute kidney injury induced by I/R also has been studied. This renal injury may be consequence of several factors as renal transplantation and involves vascular factors and tubular damage associated with high significant morbidity and mortality. Curcumin was administered orally to rats (200 mg/kg/day for 7 days) subjected to bilateral renal ischemia for 45 min followed by 24 h reperfusion [15]. Curcumin significantly attenuated the reduction of serum GPx and the levels of urea, cystatin C, and MDA in serum and the increase of the MDA concentration, nitric oxide and protein carbonyl content in kidney of rats with I/R [15]. On the other hand, Jacob et al. [41] observed, in mice with deficient immune response and glomerulonephritis injury, that curcumin administration reduces GS and improves renal function (evaluated by BUN and albuminuria), which was associated to a decrease in inflammatory markers (TGF-β and MCP-1) and matrix proteins (fibronectin, laminin and collagen).

### Shock-wave lithotripsy (SWL)

SWL is commonly used for treatment of renal stones and ROS are involved in the pathophysiology of renal injury due to SWL. The protective effect of curcumin (75 mg/kg/day for 35 days) against renal injury induced by SWL was studied in Sprague-Dawley rats [14]. Curcumin prevented interstitial, glomerular, tubular epithelial and endothelial cellular injuries by decreasing of iNOS and p65 (the active subunit of NF-κB) expression and serum nitric oxide levels. This protective effect was also associated with increased levels of GSH and attenuation of high levels of MDA in kidney [14].

### Triiodothyronine (T3)-induced renal injury

One of the most important effects of thyroid hormones (T3 and T4) is the elevation of mitochondrial respiration, producing a hyper-metabolic state with excess generation of free radicals, Thyroxine has been reported to induce renal hypertrophy with a rise in the DNA content. However, there is a paucity of information on T3-induced oxidative damage to mammalian kidney in general and with respect to antioxidant treatment in particular [66].The effect of curcumin treatment (30 mg/kg/day for 15 days) was evaluated on renal damage and oxidative stress induced by T3 administration. It was found that curcumin was able to attenuate the mitochondrial lipid peroxidation, the enhanced SOD activity and the histopathological changes secondary to T3-administration [66].

## Renal injury induced by drugs

### Cisplatin and oxaliplatin

Cisplatin is an effective anticancer drug used against lung, ovarian cancer and some lymphomas, however renal damage has limited its use. It has been well established that oxidative stress is one of the mechanisms involved in cell damage induced by cisplatin. Indeed, a decrease of antioxidant defense is clearly observed *in vivo* and *in vitro* experimental models [19]. Antunes et al. [9] reported curcumin administration (8 mg/kg before and after cisplatin injection) provided protection against cisplatin induced neurotoxicity, ototoxicity and nephrotoxicity (evaluated by serum creatinine and creatinine clearance) and oxidant stress (evaluated by MDA and GSH levels) in rats. Moreover, Kuhad et al. [45] designed a two-day curcumin pretreatment and in parallel treatment of 15, 30 and 60 mg/kg of curcumin in a model of cisplatin-induced nephrotoxicity. The cisplatin-treated group that received 60 mg/kg of curcumin showed normal renal function (evaluated by measuring urea levels and creatinine clearance), which correlated with lipid peroxidation reduction. Interestingly, curcumin administration in cisplatin-treated animals attenuated, in a dose dependent manner, the cisplatin-induced decrease in GSH, SOD and CAT [45]. In addition, Ueki et al. [82] studied the effect of curcumin administration (100 mg/kg ip) on the inflammatory mechanisms involved in the pathogenesis of cisplatin-induced renal injury in mice. Curcumin prevented cisplatin-induce tubular necrosis, decreased renal dysfunction and the increase of pro inflammatory markers including of TNF-α in serum, and TNF-α and MCP-1 in renal tissue, and a rising of intracellular adhesion molecule 1 (ICAM-1) mRNA in kidney. Oxaliplatin, another platinum-based chemotherapeutic agent can induce renal damage and oxidant stress. *In vitro* studies performed by Waly et al. [84] showed that oxaliplatin or cisplatin induced oxidative stress in human embryonic kidney cells (HEK 293). These cells also showed a decrease in total antioxidant capacity (TAC) and inhibition of the activity of SOD, CAT and GPx. Interestingly, curcumin added to these cell cultures significantly restored TAC and activity of the above mentioned antioxidant enzymes. Together, these studies clear up the ability of curcumin to decrease oxidative stress through modulation of those enzymes.

### Gentamicin

Gentamicin is an aminoglycoside used in the treatment of infections caused by Gram-negative bacteria that induces renal injury as a side effect. Curcumin treatment (200 mg/kg/day for 10 days) ameliorated the gentamicin-induced nephrotoxicity in rats [7]. Moreover, Manikandan et al. [50] observed a renoprotective effect after curcumin administration (200 mg/kg/day for 7, 15 and 30 days) in gentamicin-treated animals. Nephrotoxicity was evidenced by increased serum creatinine and BUN. An increase in ROS and renal lipoperoxidation and a reduction in GSH and in the antioxidant enzymes GPx, GST, SOD and CAT were related to the impaired glomerular filtration. The degree of these alterations was diminished in animals treated with curcumin. Furthermore, curcumin modulated the inflammatory response in gentamicin-treated rats through NF-κB expression in a time-dependent manner [50].

### Cyclosporin A (CsA)

CsA is widely used as immunosuppressant drug in organ transplantation to prevent rejection. This therapeutic agent induces renal damage as side effect. Tirkey et al. [81] showed that curcumin was effective by protecting CsA-induced damage, since animals that received the phenolic compound plus CsA did not show renal function deterioration or depletion of antioxidant enzymes. CsA increased the thiobarbituric acid reactive substances (TBARS), decreased renal endogenous antioxidant enzymes and deteriorated the renal function as assessed by increased serum creatinine, BUN and decreased creatinine and urea clearance. Curcumin reduced elevated levels of TBARS, attenuated renal dysfunction, increased the levels of antioxidant enzymes and normalized the altered renal morphology in CsA treated rats.

### Adriamycin (doxorubicin)

Adriamycin is a chemotherapeutic drug that may induce nephrotoxicity as a side effect. Treatment with curcumin (200 mg/kg/day in 1% gum acacia for 30 days) markedly protected against adriamycin-induced proteinuria, albuminuria, hypoalbuminaemia and hyperlipidemia. Furthermore, curcumin also reduced urinary levels of the enzyme *N*-acetyl-β-d-glucosaminidase (NAG), a marker of tubular damage [83].

### Chloroquine

Chloroquine is a drug used in the malaria treatment and induces renal injury and oxidant stress as secondary effect. It was found that THU (80 mg/kg/day for 15 days) and curcumin treatment prevents chloroquine-induced nephrotoxicity and lipid peroxidation and also the decrease in the antioxidants vitamin C, vitamin E, SOD, CAT and GPx in kidneys of these rats [60].

## Renal injury induced by chemicals

### Sodium fluoride (NaF)

Renal damage induced by chemicals also has drawn attention. Nabavi et al. [54] reported the renoprotective effect of curcumin (10 and 20 mg/kg) in rats with nephrotoxicity induced by NaF. Renal damage in these rats relies on an impairment of renal function evidenced by increased serum creatinine and BUN and structural impairment due to interstitial edema, inflammation and fibrosis which was associated with increased nitric oxide, peroxides, ROS and MDA. On the other hand, curcumin treated animals showed an improvement in renal function and showed less histological damage associated to restoration of SOD and CAT activities and increased GSH levels.

### Heavy metals

**Fig. 3:**
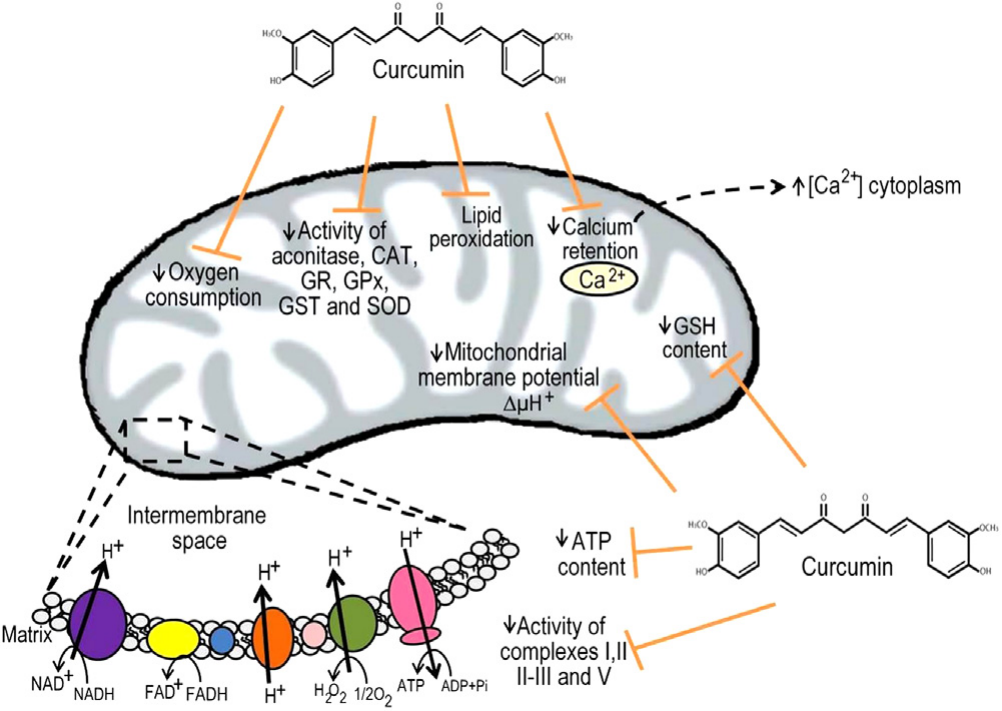
Curcumin is able to prevent mitochondrial dysfunction associated to renal injury. Curcumin is able to prevent lipid peroxidation and the decrease in the following mitochondrial determinations: oxygen consumption, activity of complexes I, II, II-III and V, activity of aconitase and antioxidant enzymes, GSH content, membrane potential, calcium retention and ATP content [51]. GSH (Glutathione), SOD (superoxide dismutase), CAT (catalase), GPx (glutathione peroxidase), GST (glutathione-*S*-transferase), GR (glutathione reductase), NAD^+^ (nicotinamide adenine dinucleotide), NADH (nicotinamide adenine dinucleotide, reduced form), FAD^+^ (flavin adenine dinucleotide), FADH_2_ (flavin adenine dinucleotide, reduced form), ATP (adenosine triphosphate), ADP (adenosine diphosphate).

Studies evaluating the effect of curcumin in experimental models of nephrotoxicity by heavy metals such as chromium (Cr) [51], cadmium [30] and mercury (Hg) [1] have been conducted. Curcumin treatment attenuated renal dysfunction, oxidative stress and the decrease in antioxidant enzymes induced by metals. Interestingly, Molina-Jijón et al. [51] demonstrated that curcumin has a protective effect against nephrotoxicity induced by hexavalent chromium (Cr VI), and this property was related to the nuclear translocation of Nrf2, prevention of oxidant stress and preservation of the activity of antioxidant enzymes and of mitochondrial function in the kidney. In this study, a pretreatment of 10-day with 400 mg/kg of curcumin attenuated the structural and functional damage to the kidney which was associated with the prevention of mitochondrial oxidant stress and of the decrease in the following mitochondrial determinations: oxygen consumption (state 3), respiratory control, ATP content, calcium retention and membrane potential. Also curcumin prevented the decrease in the following enzymatic activities: aconitase, antioxidant enzymes and mitochondrial respiratory complexes I, II, II–III and V [51], (Fig. 3). This was the first demonstration that the prevention of renal injury was associated to the preservation of mitochondrial function.

### Ferric nitrilotriacetate

Ferric nitrilotriacetate is a carcinogen and strong inductor of renal oxidative stress. The effect of curcumin and THU on ferric nitrilotriacetate induced oxidant stress in male ddY mice was studied [55]. Animals were fed along four weeks with 0.5% curcumin or 0.5% THU before the administration of ferric nitrilotriacetate. Curcumin inhibited 4-hydroxy-2-nonenal-modified protein formation and THU inhibited lipid peroxidation and renal abundance of 4-hydroxy-2-nonenal (4-HNE, a marker of lipid peroxidation)-modified proteins and 8-hydroxy-2′-deoxyguanosine (a marker of DNA damage). THU induced GPx, GST and NQO1, as well as or better than curcumin.

## Final remarks

**Fig. 4:**
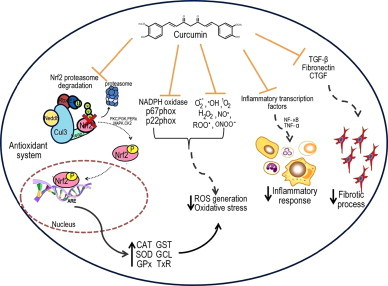
Curcumin is able to prevent several mechanisms leading to renal injury. Curcumin renoprotective effects have been associated with the prevention of three main factors, first the reduction of oxidative stress by (a) preventing the generation of O_2_^**−∙**^ and scavenging different reactive oxygen species, and (b) by preventing the Nrf2 degradation by ubiquitin proteosoma pathway, thus an increase of many antioxidant enzymes. Curcumin has been shown also to be able to reduce inflammatory process by reducing the inflammatory transcription factors such as NF- κB and TNF-α. On the other hand the reduction of cytokines such as TGF-β or CTGF eventually prevents a fibrotic process. ROS (reactive oxygen species), O_2_^-∙^ (superoxide),^∙^OH (hydroxyl radical), H_2_O_2_ (hydrogen peroxide), ONOO^−^ (Peroxynitrite), ^1^O^2^ (Singlet oxygen), NO^∙^ (Nitric oxide), ROO^**∙**^ (peroxyl radical), Nrf2 (translocation of nuclear factor erythroid-derived 2), ARE (Antioxidant responsive elements), Keap1 (Kelch-like ECH-associated protein 1), Cul3 (cullin 3), Nedd8 (Neural precursor cell expressed developmentally down-regulated 8), u (ubiquitin), SOD (superoxide dismutase), CAT (catalase), GPx (glutathione peroxidase), GST (glutathione-*S*-transferase), GCL (glutathione-cystein-ligase), TrX (thioredoxin), TGF-β (factor transforming growth beta), CTGF (connective tissue growth factor), NF- κB (nuclear factor kappa-light-chain-enhancer of activated B cells), TNF-α (tumor necrosis factor), PKC (protein kinase C), PI3K (phosphoinositol 3-kinase), PERK (protein kinase RNA-like endoplasmic reticulum kinase) MAPK (mitogen-activated protein kinase), CK2 (Casein kinase2).

According to epidemiological evidence, acute renal injury is a serious health and economical problem across the world with increasing cases since this disease can have its own etiology but also can be a complication from other diseases or can be a side effect from several medical treatments. The urgency to develop renoprotective strategies sets the eyes in compounds as curcumin, which has been used in the traditional medicine, specifically because its protective effects against renal damage. In this context, the experiments of Tapia et al. [80], in which curcumin was able to revert established renal injury and systemic alterations in rats with 5/6NX, are promising. At cellular and molecular levels, recent studies have demonstrated that this compound attenuates ROS generation and activates signaling pathways that involve the release of Nrf2 from Keap1, promoting transcription of genes that induce the expression of antioxidant system (GPx, GST, CAT, and SOD). Also, recent evidence shows that improvement of mitochondrial dysfunction induced during nephrotoxicity seems to be a key mechanism in curcumin protection and indirect reduction of ROS production through mechanisms including a decrease of O_2_^−∙^ through the down regulation of expression of some NADPH oxidase subunits such as p67phox, p22phox and NOX4 essential for O_2_^¯∙^ generation, which is useful against inflammation, a common process during kidney injury. In this regard, curcumin also diminishes the expression of NF-κB and TNF-α, TGF-β, extracellular matrix proteins collagen type IV, fibronectin and growth factors such as CTGF. These proteins are closely involved not only in inflammation but also, in the remodeling tissue which leads to renal fibrosis. Fig. 4 and Table 1 summarize some cell protection mechanisms of curcumin in renal damage. The information presented in this paper identifies curcumin as a promising renoprotective molecule against renal injury.

## Figures and Tables

**Table 1:** Factors associated to renoprotection by curcumin.

	**Studied targets**	**Effect of curcumin treatment**	**Renal injury models**
Transcription factors	Nrf2	Promotes the Nrf2 translocation to the nucleus, the major regulator of the antioxidant response	5/6 NX, HM (Cr VI)
Pro-oxidant enzymes	NADPH oxidase subunits: Nox4, p67phox, p22phox	Attenuates oxidative stress by reducing levels of subunits of NADHP oxidase	Diabetic nephropathy
Antioxidants	GPx	Increases the activity of antioxidant enzymes	Diabetic nephropathy, 5/6 NX, I/R,SWL,T3, Cisplatin, Gentamicin, CsA, Chlr, NaF, HM (CrVI), FNT
		Increases the synthesis and concentration of GSH	
	CAT		
	GR		
	GST		
	SOD		
	NQO1		
	GSH levels		
Profibrotic cytokines	VEGF	Attenuates the expression of the cytokines promoting a decrease in the inflammatory response	Diabetic nephropathy, I/R
	TGF-β		
	CTGF		
	Osteopontin		
Extracellular matrix protein	Fibronectin	Promotes a decrease in matrix proteins	I/R
	Collagen IV		
	Laminin		
Pro-inflammatory mediators	TNF-ɑ	Reduces the inflammatory response	Diabetic nephropathy, 5/6 NX, I/R, SWL, Cisplatin, Gentamicin
	MCP-1		
	NF-κB		
	p65 (NF-κB subunit)		
	JNK/NF-κB		
	COX-2	Decreases the inflammatory markers by blocking its overexpression	
	iNOS		
	IL-1β		
	PLP2		
	TGF-β		
Mitochondrial function markers	Oxygen consumption	Prevents the decrease of mitochondrial parameters	HM (Cr VI)
	ATP content		
	Calcium retention		
	Mitochondrial membrane potential	Protective effect associated with the preservation of mitochondrial function	
	Activity of mitochondrial respiratory complexes		

5/6NX:5/6 nephrectomy, I/R:ischemia and reperfusion, SWL: shock-wave lithotripsy, T3: triiodothyronine, CsA: cyclosporine, Chlr: chloroquine, NaF: sodium fluoride, HM: heavy metals, FNT: ferric nitrilotriacetate.
